# Nexuses between crude oil imports, renewable energy, transport services, and technological innovation: a fresh insight from Germany

**DOI:** 10.1007/s13202-022-01487-0

**Published:** 2022-03-31

**Authors:** Zhang Yu, Hafiz Muhammad Zia-ul-haq, Ateeq ur Rehman Irshad, Muhammad Tanveer, Kiran Jameel, Laeeq Razzak Janjua

**Affiliations:** 1 School of Economics and Management, Chang’an University, Xi’an, China; 2 Department of Business Administration, ILMA University, Karachi, Pakistan; 3 Faculty of Business Economics and Social Development, Universiti Malaysia Terengganu, Kuala Terengganu, Malaysia; 4 Department of Mathematics and General Sciences, Prince Sultan University, Rafah Street, Riyadh, 11586 Saudi Arabia; 5 Prince Sultan University, Rafah Street, Riyadh, 11586 Saudi Arabia; 6 College of Business and Management, Institute of Business and Management, Karachi, Pakistan; 7 Poznan University of Economics and Business, Poznan, Poland

**Keywords:** Crude oil, Renewable energy, Trade, Transport services, Industrial value-added

## Abstract

This research attempts to model the association of crude oil imports with several macroeconomic factors such as renewable energy, transport services, trade, industrial value-added, and patents, using Germany’s annual data covering the period of 1990–2020. Employing the Autoregressive Distributed Lag model, this study finds a significant co-integration relationship among targeted variables. Moreover, this study provides empirical evidence on the influence of given macroeconomic factors in determining crude oil imports of Germany. Results reveal that transport services and industrial value-added positively and significantly influence crude oil imports in the long and short run. Similarly, trade is discovered to have a significant positive impact on oil imports only in the long run. In contrast, findings reveal a significant negative association of renewable energy with crude oil imports. Hence, this research implies that the transportation sector and industrial production strongly depend on crude oil consumption. At the same time, promoting renewable energy in these segments could significantly help economies control crude oil demand and achieve sustainability by reducing the economic burden and protecting the environment.

## Introduction

Business and industries are indispensable to stable provision and pursuing evolution that has already started from alternate energy solutions. This does not facilitate the transport sector alone but also exploits the economic efficiency of energy and petroleum products. The petroleum industry consisted of refining crude oil, processing natural gas, and converting many products. The process is based on operations of oil and gas fields, gas plant operations, refining the raw oil and refinery operations, and technologies used while refining them (Kanaboshi et al. 2021). The Petroleum industry is a sought-after in the global economy as a primary source of fuel consumption (Solaymani and Kari 2013). It comprises a wide assortment of uses ranging from the main generation of energy and transportation fuels (Lin and Yousaf 2020). Consequently, this industry possess powerful widows in the world’s economy, and any fluctuation in the oil prices have a great reflection in term of pricing in a maximum of the manufacturing, transport sector and ultimately consumers (Pablo-Romero et al. 2017).

According to OPEC, the worldwide demand for crude oil in 2020 fell to 91 million barrels a day due to the closure of economic mobility, production, and operational activities during the lockdown. However, the persistent trend is forecasted to increase in 2021 from 96.6 mills. Barrels per day (OPEC 2020). Although oil is an integral substance used in different applications, the transport sector is the largest oil consumer globally and accounts for 1/3rd of the global oil demand. The transport sector accounts for 20% of the world’s energy users (Capuano 2018). All the means of transportation, including roads, aviation, and shipping, are directly or indirectly linked with oil consumption as an integral substance (Khan et al. 2022a). Besides, in 2045, the contribution of transportation fuels, i.e., gasoline and diesel, is projected to remain the leading consumed product for 109 mills. barrels per day (Nouni et al. 2021). Due to heavy dependence on oil products, transport is critical for polluting the environment while having a heightened level of CO_2_ emission and its adverse effects on urban inhabitants (Kanaboshi et al. 2021). Usage of energy has a positive correlation with CO_2_ emission; however, the substance of clean energy reduces the level of CO_2_ emission (Chen et al. 2020). These stimulate pollutant in the form of toxic emission and produce hazardous waste that is released into the atmosphere (Khan et al. 2022a).

Being the largest petroleum industry in the EU, the German petroleum industry is critical to energies business growth while maintaining CO_2_ emission. Germany stood at 2nd CO_2_ emitter economy in EU (Janssens-Maenhout et al. 2017). However, the EU's total 26% CO_2_ emissions were from the transport sector (Sajid et al. 2019). Transport is the largest consumer of petroleum products in Germany, and this sector accounts for 33% in terms of energy usage and 20% in CO_2_ emission (Haasz et al. 2018). Germany's energy consumption is likely to increase nearly 3%, and the energy-based CO_2_ emission in Germany is stood at a 4% increase in 2021, mainly due to the economic activities after COVID-19 (Chen et al. 2020; Yu and Khan 2021a). However, pandemic-2020 has been a great setback for the industry as mobility is restricted across the world. That ultimately curtailed the demand of the largest oil-consuming sector, and hence, the oil benchmark touched its lowest level in April 2020.

All the available literature so far have studied crude oil production, consumption, and its effect on the environment (Janssens-Maenhout et al. 2017; Khan et al. 2022a; Kanaboshi et al. 2021; Khan et al. 2022b; Khan et al. 2021c; Lin and Yousaf 2020; Khan et al. 2021b; Magazzino et al. 2020). But how can the policymakers make a strategic decision while keeping in mind the heightened trend of crude oil trade and its usage for mobilizing economic activities that ultimately industrialized the product while using the renewable energy alternatives? Hence, it is a matter of concern for the researcher and policymakers to make the transport future proof policies that reflect economically industrial value addition, ecologically usage of renewable energy, and socially compatible during the transformation period. Germany needs an economical, reliable, affordable, and environmentally friendly energy supply. The careful usage of energy resources that require climate protection is also a concern for the transport sector. Germany can be considered a leading market innovator for sustainable mobility solutions. These parameters of Germany enforce the researchers to critically analyze the association between crude oil, transportation services, renewable energy, international trade, patents, and industrial value-added.

## Literature review and hypothesis development

### Crude oil and economic

Natural resources, such as oil, minerals, and agriculture, are recognized as valuable blessings that can significantly contribute to the country's economic growth. After World War II, oil has become the dominant energy resource, particularly for transportation and manufacturing, and has been significant in developed and developing economies (Su et al. 2021). The traditional perception that a country with abundant natural resources will have faster economic growth has been challenged in the literature. The dilemma about whether resources are a curse or a blessing has been studied and investigated for decades (Gu et al. 2020; Naseer et al. 2020; Yang et al. 2021). The abundance of natural resources is inversely related to economic growth (Gylfason 2001; Khan et al. 2021a; Yu and Khan 2021b).

Crude oil has the leading position in the commodities sector, contributing to above 40% of global energy consumption. Oil price volatility is a source of concern for economies, specifically oil-dependent ones. The uncertainty has negative and disruptive economic consequences that might become an obstacle to future sustainable economic growth (Ebrahim et al. 2014; Su et al. 2021). Crude crisis including the Arab oil embargo (1970), the Iran–Iraq dispute (1980), the first Gulf war (1990), the global depression economic crisis (2008), and the current pandemic COVID-19 (2020) intensify the unpredictability of oil prices, therefore play a significant effect in countries' economic growth. Gold, on the other hand, serves as a store of value, a safe haven, or an economic hedge against rising commodity instability (Wang et al. 2021a, b).

Even though oil is consumed in a wide range of applications, the transportation sector is the world's largest oil user, accounting for 1/3 of global oil demand. The transport sector accounts for 20% of the world's energy users (Capuano 2018). Haasz et al. (2018) addressed the EU-28's transportation sector's decarbonization prospects. They observed that crude oil affected the transportation sector positively, meanwhile consuming 33% of all energy in the EU. Khan et al. (2022a) claimed that the utilization of oil as an integral substance seems to be directly or indirectly linked to all modes of transportation, including roadways, airplanes, and shipping. However, the expansion of all modes of transportation could lead to an increase in crude oil consumption. Additionally, transportation fuels, such as gasoline and diesel, are anticipated to be the most widely consumed product in 2045, accounting for 109 million barrels per day (Nouni et al. 2021).

As transport flows continue to expand, the development of robust models for forecasting future trade flows has become more critical (Babri et al. 2017). The utilization of crude oil is traced through inter-regional trade from the sources of exploitation to the sources of final use. Wu and Chen (2019) studied crude oil usage in inter-regional trade. They found that crude oil consumption is traced through inter-regional trade networks from the sources of exploitation to the sinks of final use. The top five oil consumers, the USA, Mainland China, Japan, South Korea, and Canada, account for half of world oil consumption. The worldwide amount of oil reflected in trade is 20%, and non-oil trade plays a comparable role in the international oil flow as direct oil trade.

International competitiveness for crude oil has escalated in the last decade with industrializing countries like China and India (Khan et al. 2021c; Yang et al. 2015). Despite this oil, the sector is one of the world's largest and most capital-intensive sectors (Khan et al. 2021d; Babri et al. 2017).

Sodeyfi and Katircioglu (2016) investigated the empirical analysis of oil and real GDP and industrial value addition in the Eurozone, the European Union, Latin America and the Caribbean, South Asia, and Sub-Saharan Africa. They revealed that long-run equilibrium correlations exist between real GDP, oil prices, and industrial value-added in all regions. The findings show that oil has a long-run negative impact on actual income, while the industry has a positive effect on oil.

Hence, following hypotheses have been proposed based on the previous studies;

#### H1

Crude oil and transportation services have a positive relationship.

#### H2

Trade and crude oil have a positive association.

#### H3

Industrial value-added is interlinked with crude oil.

### Crude oil and environment

Dependence on crude oil products contaminates the environment, generates a high amount of CO_2_, and has harmful effects on urban residents (Kanaboshi et al. 2021). Besides, crude oil contributes to pollution in the form of toxic gases and hazardous waste, which would then be emitted into the atmosphere (Khan et al. 2022a). The CO_2_ emissions are inversely proportional to crude oil consumption. On the other hand, renewable energy seems to have the ability to lower CO_2_ emissions (Khan et al. 2021g; Chen et al. 2020). The behavioral component is critical for considering mitigation methods for transportation energy-related CO_2_ emissions (Khan et al. 2021h; Schäfer 2012). However, behavioral changes are still difficult to simulate using energy-economy-environmental-engineering (E4) models. Some attempts have been made to bridge this gap by including behavioral characteristics in integrated energy and transportation models (Khan et al. 2021e, f; Venturini et al. 2018).

In recent years, there has been an increase in the consumption of renewable energy sources, prompting a field of knowledge into the factors that influence renewable energy usage (Chen et al. 2021). Despite previous studies that looked into the relationship between crude oil and renewable energy, however, this study tried a different approach while attempting to study the crude oil trade and its usage for mobilizing economic activities that ultimately industrialized the product while using the renewable energy alternatives.

Seriño (2019) found a negative association between renewable energy and crude oil. However, a robust belief that advancement in renewable energy sources ultimately diminishes the economy's reliance on crude oil and related products. They further argued that CO_2_ emissions as a result of a high reliance on crude oil consumption also push developing countries to use renewable energy alternatives.

Papie et al. (2018) investigated the components that contribute to the EU's growing interest in renewable energy and reflect that the EU's economy is heavily reliant on energy imports. They argued that environmental degradation (CO_2_ emissions) and oil imports forced countries to switch toward renewable energy. However, since the economy relies on renewable energy, crude oil demand will decline.

Ferrer et al. (2018) explored that reliance on crude oil usage as energy has dropped while raising the need to compare renewable energies as alternatives. Consequently, both developing and developed economies are constantly working to employ more renewable alternatives to oil.

On the basis of previous literature, following hypothesis has been developed;

#### H4

Renewable energy with crude oil is negatively correlated.

## Research methodology

### Data and variables

This study examines the influence of various economic factors in determining Crude-Oil Imports of Germany. Specifically, factors such as trade, renewable energy (RE), industrial value-added (IND), transport services (TS), and patent applications (PA) are included in the model. This study used annual crude oil imports as an outcome variable. Trade is measured as a total yearly trade volume and industrial value-added as a percentage of gross domestic product among the explanatory factors. Similarly, RE is captured in terms of its annual consumption, whereas TS is measured as a percentage of total services imports. Lastly, the total number of patent applications in a year is used to calculate the variable of PA.

Next, this study collected time series data of Germany covering a period of 1990–2020 from various databases. For instance, data on renewable energy usage are collected from International Energy Agency (IEA). Likewise, information on patent applications is collected from World Intellectual Property Organization (WIPO). In contrast, data relating to remaining variables such as trade, transport services, industrial value-added, and crude oil is collected from the World Development Indicators (WDI) database. Table 1 presents a detailed description of variables and their data sources.

**Table1:** Variable and source (period 1990–2020)

Variable names	Variable description	Source
Crude-oil	Total crude oil imports	WDI
Trade	Total trade (% of GDP)	WDI
RE	Total renewable energy used	IEA
IND	Industrial value added (% of GDP)	WDI
TS	Transport services (% of service exports, BoP)	WDI
Patent	Total patent applications	WIPO

**Fig. 1:**
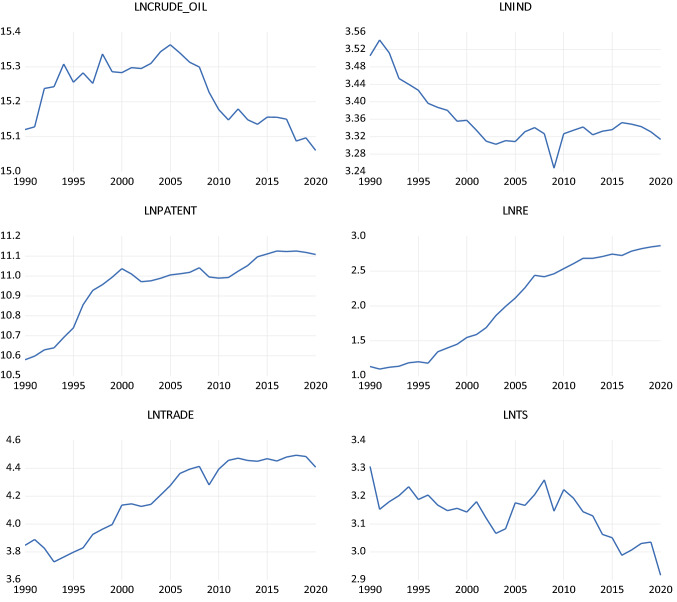
Variable patterns over the time

Further, this study also presents the trend pattern of the variables in Fig. 1 to understand the variables better. Figure 1 displays the trend pattern of all related variables from 1990 to 2020. It can be viewed that all variables are transformed into natural logarithm values to tackle issues of structural breaks and data normality.

### Econometric model

This study develops its econometric model, mentioned in Eq. 1, to analyze the research objective. Equation 1 constitutes regressing crude-oil on RE usage, trade, industrial value-added, transport services, and patent applications. Employing time series analysis, this study estimated the given econometric model for the case of Germany to assess hypothesized influence of factors such as RE usage, trade, industrial value-added, transport services, and patents on the volume of crude-oil import. Moreover, this study also took the natural logarithm of the variables in the given equation for data smoothening.

1 $${\text{LNcrude}} - {\text{oil}}_{t} = \beta_{0} + \beta_{1} {\text{LNRE}}_{t} + \beta_{2} {\text{LNT}}_{t} + \beta_{3} {\text{LNIND}}_{t} + \beta_{4} {\text{LNTS}}_{t} + \beta_{5} {\text{LNPA}}_{t} + \varepsilon_{t}$$

In the above equation, LN denotes natural logarithm, whereas $\beta$ and *ε* represent the coefficient and error term of the model, respectively. Next, crude-oil represents the volume of crude-oil imports; *T* represents trade volume; RE represents renewable energy usage, IND represents the industrial value-added; TS represents transport services; and PA represents the total number of patent applications. Lastly, the time trend is denoted by the expression “*t*”.

### Data analysis

#### Unit root test

While conducting time series analysis, assessment of the stationarity is considered a crucial step that can cause serious complications. Therefore, this study also evaluated the stationarity of the data in the initial stage of analysis. For this purpose, two most commonly used unit root tests are employed to assess the stationarity of the variables. These tests include Augmented Dickey–Fuller (ADF) (1979) test and Phillips–Perron (1988) test. This study estimated Eq. 2 for the ADF test to analyze its null hypothesis that “time series is non-stationary.” Likewise, Eq. 3 is estimated to analyze the null hypothesis of the Phillips–Perron test, which states “the existence of unit root” in the data. Hence, accepting alternative hypotheses in both cases demonstrates the suitability of the data for time series analysis. Equations 2 and 3 are given below to present specifications of the ADF and Phillips-Perron test, respectively.

2 $$\Delta Y_{t} = c + \beta_{t} + \delta Y_{t - 1} + \mathop \sum \limits_{i = 1}^{k} \alpha_{i} \Delta Y_{t - 1} + \varepsilon_{t}$$

3 $$\Delta Y_{t} = \beta_{t} + \pi Y_{t - 1} + u_{t}$$

Here, in the given equations, “*Y*_*t*_” and “*Y*_*t−*1_” denote variables to be examined in current period “*t*” and their previous annual values from the period “*t−*1”, respectively. Similarly, “*c*” is an intercept and “∆” represents first difference operator. Next, coefficients of time trend and lagged variables are represented by “*β*” and “$\delta$”, respectively. While “*π*” shows the length of lags in Eq. 3. In the end, error terms in Eqs. 2 and 3 are denoted by “*ε*” and “$u$”, respectively.

#### Autoregressive distributed lag model (ARDL) co-integration technique

Once stationarity of the data was assessed and proved, this study applied Autoregressive Distributed Lag (ARDL) Model approach to examine co-integration among targeted variables included in the model such as crude-oil, RE usage, trade, IND, TS, and PA. ARDL is the most widely adopted technique to conduct time series analysis because of its appropriateness and relative advantages over other econometric approaches. First, ARDL estimates are relatively less affected by the issue of stationarity in the data and small sample sizes. Similarly, it has the advantage of adequately analyzing both short and long-term relationships among variables. Therefore, the following ARDL model is developed in Eq. 4 to analyze specific hypothesized relationships between crude-oil and other economic factors of this study.

4 $$\begin{aligned} \Delta {\text{LNCrude}} - {\text{oil}}_{t} = & \beta_{0} + \beta_{1j} \Delta {\text{RE}}_{t - j} + \beta_{2i} \Delta {\text{Trade}}_{t - j} + \beta_{3j} \Delta {\text{IND}}_{t - j} \\ & + \beta_{4} \Delta {\text{TS}}_{t - j} + \beta_{5j} \Delta {\text{PA}}_{t - j} + \gamma_{1} {\text{LRE}}_{t - 1} + \gamma_{2} {\text{Trade}}_{t - 1} \\ & + \gamma_{3} {\text{IND}}_{t - 1} + \gamma_{4} {\text{TS}}_{t - 1} + \gamma_{5} {\text{PA}}_{t - 1} + \varepsilon_{t} \\ \end{aligned}$$

The developed ARDL model represents long-term and short-term coefficients by ${"\beta }_{1}"$ to “${\beta }_{6}$” and “${\gamma }_{1}$” to “${\gamma }_{6}$”, respectively. Under the ARDL approach, this study determined co-integration by examining “no co-integration” as a null hypothesis against the existence of co-integration. Specifically, *F*-test is used to investigate the null hypothesis by comparing *F*-statistics with critical values. The null hypothesis of no co-integration is accepted if the *F*-statistic is less than the lower critical limit. In contrast, the null hypothesis is rejected if the *F*-test statistic is greater than the upper critical value. Further, the *F*-statistic falling within two key bounds results in inconclusiveness. Hence, accepting the null hypothesis means that long-term coefficients for all six independent variables are equal to zero, indicating that there is no co-integration among variables. On the other hand, if the null hypothesis is rejected, it proves that long-term coefficients are not equal to zero, thus, indicating the existence of significant co-integration among variables. In contrast, the significance of individual coefficient values helps in hypothesis testing relating to the influence of specific explanatory factors on crude-oil imports in both long and short terms separately.

#### Diagnostic tests and model stability

In the end, this study also conducted diagnostic testing to ensure the accuracy of the results. Notably, the problems of serial correlation, normality, and heteroscedasticity are assessed in the estimated model. Hence, the Breusch–Godfrey LM test is employed to assess serial correlation in the error term. Similarly, heteroscedasticity is examined by employing the Breusch–Pagan test. Next, normality is assessed using the Jarque–Bera test. In addition, this study also determined the model’s stability by employing cumulative sum (CUSUM) and cumulative sum of square (CUSUMSQ).

## Results and discussion

Table 2 presents descriptive statistics related to Germany for the variables of this study. All variables were transformed into a natural logarithm to tackle structural breaks and data normality problems. Particularly, this study provides mean, median, maximum, minimum, standard deviation, skewness, and kurtosis values for 31 observations of each variable. Mainly, results of mean, minimum, and maximum values are specified i.e., crude-oil (15.22, 15.06, 15.36), lnRE usage (2.019, 1.094, 2.865), trade (4.195, 3.727, 4.493), IND (3.363, 3.247, 3.541), TS (3.137, 2.916, 3.306), and PA (10.952, 10.579, 11.125). Further, descriptive results show that the variables of IND and PA are highly skewed toward the right and left sides, respectively. At the same time, TS is found moderately skewed left. However, the remaining variables of crude-oil, RE, and trade are found to have approximately symmetric distributions.

**Table 2:** Descriptive statistics

Statistics	LnCrude_oil	LnIND	LnPA	LnRE	LnTrade	LnTS
Mean	15.22654	3.3630	10.9529	2.0197	4.19522	3.1374
Median	15.2432	3.3406	10.9951	2.1137	4.27553	3.1525
Maximum	15.3635	3.5416	11.1257	2.8651	4.49350	3.3061
Minimum	15.0605	3.24788	10.5797	1.0948	3.72754	2.9163
Std. dev	0.0874	0.06672	0.16645	0.6644	0.26553	0.0852
Skewness	− 0.21165	1.2249	− 1.1434	− 0.14506	− 0.42085	− 0.59073
Kurtosis	1.73091	3.9495	3.05573	1.3762	1.6414	3.1275
Observation	31	31	31	31	31	31

Next, the Pearson correlation matrix is presented in Table 3 to assess bivariate correlation among different variables of this study. It is shown that crude-oil import negatively correlates with industrial value-added, patent applications, RE usage, and trade. However, transport services are found to have a positive bivariate correlation with crude oil import. This positive correlation can be justified because improvements in transport services ultimately enhance the transportation industry, thus increasing crude-oil consumption in a country.

**Table 3:** Correlation matrix

Variable	LnCrude_oil	LnIND	LnPA	lnRE	LnTrade	LnTS
LnCrude_oil	1					
LnIND	− 0.11722	1				
LnPatent	− 0.13629	− 0.86701	1			
lnRE	− 0.47176	− 0.703239	0.828811	1		
LnTrade	− 0.38489	− 0.72104	0.86330	0.97408	1	
LnTS	0.45293	0.40634	− 0.62038	− 0.58204	− 0.523645	1

Next, this study performed a unit root analysis to assess the issue of stationarity in the data. The ADF test and Philips-Perron test have been applied to check stationarity in the variables. The results of the ADF test and Philips-Perron test are reported in Table 4. All variables are found significant, and results from both tests are completely consistent. Specifically, crude-oil and TS are proved stationary at level (I (0)), whereas results indicate that all variables are including RE usage, trade, IND, and PA, are also found stationary at the first difference (I (1)). Employing the ARDL technique requires that all variables must be stationary at level (I (0)) or first order (I (1)). These unit root results prove that none of the data series are second order (I (2)). Hence, findings fully support applying the ARDL technique to analyze hypothesized relationships of this study in both long-run and short-run.

**Table 4:** Unit root

Variable	Augmented Dicky–Fuller			Phillips–Perron		
	Level	First difference	Order	Level	First difference	Order
LnRE	− 1.4387 (0.7469)	− 4.3685 (0.0692)*	I(1)	− 1.3356 (0.73612)	− 4.3477 (0.0144)*	I(1)
LnIND	− 0.2126 (0.9636)	− 4.0966 (0.0137)***	I(1)	− 0.6543 (0.0748)	− 4.2491 (0.0478)***	I(1)
LnPatent	− 1.5423 (0.1839)	− 7.0193 (0.000)***	I(1)	− 1.4783 (0.53699)	− 7.4711 (0.0000)***	I(1)
LnCrude_Oil	− 3.3613 (0.0238)**	− 6.1163 (0.0000)***	I(0)	− 3.2148 (0.1244)**	− 7.7117 (0.0000)***	I(0)
LnTrade	− 1.0045 (0.6664)	− 0.3760 (0.000)***	I(1)	− 1.9870 (0.311)	− 0.7819 (0.000)***	I(1)
LnTS	− 2.6483 (0.0870)*	− 1.6967 (0.000)***	I(0)	− 2.4744 (0.0814)*	− 2.3248 (0.0001)***	I(0)

Then, this study applied ARDL bound approach to examine the existence of a co-integration relationship among variables. The *F*-test is employed to test the null hypothesis of no co-integration. The findings from the *F*-test are provided in Table 5. It is found that estimated *F*-statistics 16.10 is greater than upper critical values of 4.15, 3.38, and 3 at 1%, 5%, and 10% level of significance. Hence, the null hypothesis of no co-integration is rejected. This strongly indicates that there exists a significant co-integration relationship among variables of this study, including crude-oil, RE, trade, IND, TS, and PA.

**Table 5:** Result of ARDL co-integration

*F* statistics	Significance level	Critical value for bound test	
		Lower bound	Upper bound
16.10091, *K* = 5 (lag:1, 3,3,3,2,2)	10%	2.08	3
	5%	2.39	3.38
	2.5%	2.7	3.73
	1%	3.06	4.15

After the co-integration relationship is proved, this study conducted short-run and long-run regression analysis using the ARDL model approach. The ARDL technique of short-run and long-run analysis that tackles the simultaneous effect of explanatory variables in a multivariate setting is considered suitable for investigating the one-to-one relationship of each explanatory factor with the dependent variable. This study provides findings of ARDL estimations in Table 6. Results reveal that TS has a positive and significant influence on crude-oil imports at a 5% level in the short and long terms. Specifically, a 1% improvement in TS enhances crude-oil imports by 0.44% and 0.21% in the long and short-run, respectively. This indicates that an efficient transport sector significantly enhances crude-oil consumption in an economy. This finding is supported by the previous studies (Nouni et al. 2021; Capuano 2018; Khan et al. 2022a), which argued that crude-oil consumption is strongly associated with various modes of transportation.

**Table 6:** Estimated long run and short run estimations

Dependent variable lnCrude oil, (lag:1, 3,3,3,2,2)							
Long run				Short run			
Variable	Coefficient	*T*-ratio	*P*-Value	Variable	Coefficient	*T*-ratio	*P*-Value
LnPatent	0.316637	1.2385	0.2705	ΔLnPatent	0.0004	0.0047	0.9812
lnTS	0.44708	2.6656	0.0448**	ΔlnTS	0.2126	3.77228	0.0130**
lnTrade	0.6590	2.5883	0.0490**	ΔlnTrade	0.1839	1.82857	0.1261
lnRE	− 0.4843	− 5.5556	0.0024***	ΔlnRE	− 0.2593	− 5.32968	0.0031***
lnIND	2.0340	5.1482	0.0036***	ΔlnIND	0.10004	0.57263	0.0917*
Constant	25.20375	7.4787	0.0007***	**ECT(**− **1)**	− 0.56996	− 15.2769	0.0002***

R-square 0.9110, Adjusted R-square 0.8437

Bold shows the ECT

***indicate 1%, **indicate 5% and *indicate 10% level of significance

Additionally, results also reveal that RE usage has an inverse and significant effect on crude-oil import at a 1% level in both long and short-run analysis. It is precisely found that a 1% increase in RE usage decreases crude-oil import by 0.48% and 0.25% in the long-run and short-run, respectively. This implies that promoting renewable energy sources in an economy could significantly reduce the consumption of traditional energy sources such as crude oil. This finding also concurs with the existing literature; for instance, Seriño (2019) argued that an economy could decrease its dependence on crude-oil consumption by adopting renewable energy sources. Likewise, Papie et al. (2018) also stated that RE usage is strongly associated with a country’s dependence on energy imports. Notably, oil imports push such economies to focus on RE usage. In turn, with more reliance on RE sources, crude-oil demand ultimately declines in an economy.

Next, it is also discovered that IND positively influences crude-oil import at a 1% level of significance in the long run. Similarly, the short-run coefficient of IND is also found positive and significant at the 10% level. The results indicate that a 1% rise in IND would significantly enhance crude oil import by 2.03% and 0.10% in the long-run and short-run, respectively. This implies that countries with a strong dependence on traditional energy sources face a significant increase in oil demand with more industrial production. This is consistent with the previous literature, which provided a positive impact of industry on demand for oil in a country (Sodeyfi and Katircioglu 2016).

Furthermore, results also illustrate that trade has a positive and significant influence on crude-oil import only in the long run. Specifically, crude-oil import is found to be increased by 0.65% in the long run due to a 1% increase in trade volume. On the other hand, trade is found to have an insignificant effect on crude oil in the short run. Likewise, the number of patent applications is also found to have an insignificant influence on crude-oil import in both the long-run and short-run. The positive and significant coefficient of trade, in the long run, implies that increasing trade performance brings valuable resources and capital to the economy, which ultimately boosts economic operations, thus, increasing the demand for crude oil. Moreover, the significant dependence of trade activities on the logistics industry ultimately enhances transport consumption in an economy. Consistent with these findings, Wu and Chen (2019) also found that oil consumption is significantly associated with inter-regional trade, and non-oil trade plays an explicitly crucial role in the international oil flow.

Hence, this study mainly provides empirical evidence on the significant association of transport services and RE usage with crude-oil imports of Germany. It is argued that improvement in transportation services significantly increases a country’s crude-oil consumption. On the other hand, promoting RE usage in an economy can significantly reduce crude-oil demand and consumption. Based on these opposite effects, this study implies that authorities should focus on bringing efficiency in the transportation sector to reduce its crude oil dependence. Moreover, promoting renewable energy usage in the transport sector could be an effective strategy for the countries to control their crude-oil consumption, which will ultimately help in achieving sustainable development goals. Existing literature also highlights the transport sector as one of the highest consumers of energy (Haasz et al. 2018). Similarly, Khan et al. (2022a) also stated that oil utilization is strongly associated with several modes of transportation such as shipping, air transportation, road transportation. Hence, considering this substantial dependence of the transport sector on energy, the authorities must promote renewable energy in vehicles, which will reduce oil consumption, thus, easing the economic burden and improving environmental performance.

Lastly, Table 7 provides the results of various diagnostic tests conducted in this study. Reported results show that *p*-values of all three tests, i.e., Breusch–Godfrey test, Breusch–Pagan test, and Jarque–Bera test, are greater than 0.05, which indicates that null hypotheses of no serial-correlation, homogeneity, and normality are accepted, respectively. This implies that the model of this study is correctly specified with normally distributed residuals having no serial correlation and heteroscedasticity.

**Table 7:** Diagnostics tests

Test	*F* statistics and *P*-value
A) Breusch–Godfrey serial correlation LM test	*F* statistics = 1.782, *P*-value = 0.367
B) Normality	Jarque–Bera1.14774, *P*-value = 0.3416
C) Heteroscedasticity test	F statistics = 2.4487, *P*-value = 0.61213

**Fig. 2:**
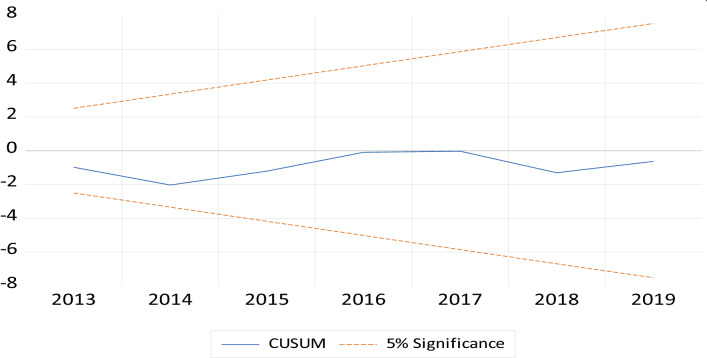
Cumulative sum (CUSUM)

**Fig. 3:**
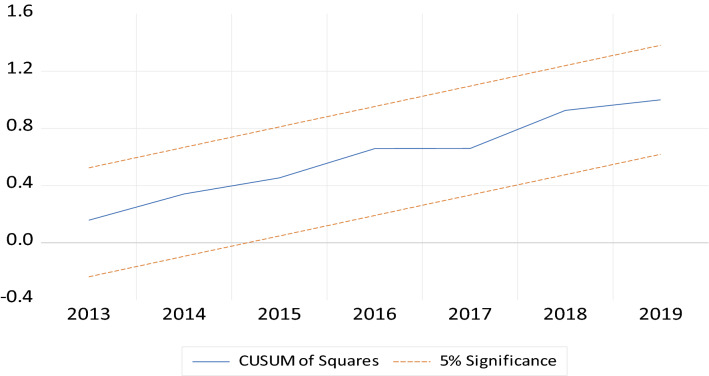
Cumulative sum of square (CUSUMSQ)

Additionally, the model’s stability is also determined in this study. Figures 2 and 3 present plots of CUSUM and CUSUMSQ; it can be viewed that both plots fall between critical limits of 5% significance level, hence, indicating that the estimated model of this study is stable and accurate.

## Conclusion and policy implications

Rising apprehensions concerning environmental adversities and resource insecurity have raised global authorities' severe concerns. The traditional energy sources and their consumption have mainly gained great attention from researchers. In this regard, crude oil is considered the most crucial asset that provides the major portion of energy around the globe. Globally, researchers have been looking to explore and understand various factors associated with crude-oil demand and import. Therefore, this study also investigates the role of various important factors that play a key role in determining the volume of crude oil imports in Germany. Mainly, this research provides empirical evidence on the association of crude-oil import with RE usage, transport services, trade, industrial value-added, and patents. The findings from econometric analysis prove the existence of a co-integration relationship among variables of this study. Specifically, results indicate a positive and significant influence of transport services and industrial value-added on crude-oil imports in long and short-run analyses. Similarly, trade has a significant and positive association with crude-oil import only in the long run. On the other hand, results prove a significant and inverse association of RE usage with the crude-oil imports of Germany. Hence, it is implied that the transportation sector and industrial production, two major consumers of crude oil, play a significant role in determining the crude oil import volume. Meanwhile, this study highlights RE usage as an effective strategy to control crude-oil consumption toward improved sustainability.

In light of rising environmental concerns and resource inefficiency, it has become critical for the authorities to devise suitable strategies to rule-out traditional dependence on imported crude oil. Therefore, this research mainly implies that authorities are needed to focus on adopting renewable energy as an alternative to conventional crude-oil consumption to reduce economic burden and enhance resource efficiency. Specifically, it is suggested to promote renewable energy in the transportation sector and industrial production because of their significant dependence on crude oil. Likewise, this study also recommends facilitating foreign inflows of advanced technology relating to renewable energy by liberalizing specific trade barriers. For instance, importing electric and hybrid technology-based vehicles could significantly reduce the transport sector's dependence on crude oil. Moreover, authorities should also emphasize importing alternative energy sources such as geothermal energy and hydroelectric power to reduce crude-oil demand. These strategies would ultimately help countries to enhance resource efficiency and reduce economic expenditures, thus, boosting economic growth.

As part of limitations, this research only incorporates data from Germany; therefore, the model can be replicated by targeting a wider group of countries to provide more generalized results. In this regard, future studies can also compare developed and developing nations to understand and analyze their dependence on crude oil. Likewise, this paper is limited to macroeconomic aspects of an economy, whereas crude-oil consumption is also linked to public perception and routine. Therefore, future studies can also incorporate public-specific factors to explore their influence on the demand for crude oil. Lastly, future studies should also incorporate multiple econometric techniques to ensure the robustness of the results better.
